# Characterization of the Chloroplast Genome Sequence of *Acer miaotaiense*: Comparative and Phylogenetic Analyses

**DOI:** 10.3390/molecules23071740

**Published:** 2018-07-17

**Authors:** Jiantao Zhao, Yao Xu, Linjie Xi, Junwei Yang, Hongwu Chen, Jing Zhang

**Affiliations:** 1 College of Horticulture, Northwest A&F University, Yangling 712100, China; jiantao.zhao@inra.fr (J.Z.); 15829031947@163.com (L.X.); 15829316842@163.com (J.Y.); 2 College of Forestry, Northwest A&F University, Yangling 712100, China; xuyaofeimeng@163.com

**Keywords:** chloroplast genome, *Acer miaotaiense*, phylogeny, microsatellite

## Abstract

*Acer miaotaiense* is an endangered species within the Aceraceae family, and has only a few small natural distributions in China’s Qingling Mountains and Bashan Mountains. Comparative analyses of the complete chloroplast genome could provide useful knowledge on the diversity and evolution of this species in different environments. In this study, we sequenced and compared the chloroplast genome of *Acer miaotaiense* from five ecological regions in the Qingling and Mashan Regions of China. The size of the chloroplast genome ranged from 156,260 bp to 156,204 bp, including two inverted repeat regions, a small single-copy region, and a large single-copy region. Across the whole chloroplast genome, there were 130 genes in total, and 92 of them were protein-coding genes. We observed four genes with non-synonymous mutations involving post-transcriptional modification (*matK*), photosynthesis (*atpI*), and self-replication (*rps*4 and *rpl*20). A total of 415 microsatellite loci were identified, and the dominant microsatellite types were composed of dinucleotide and trinucleotide motifs. The dominant repeat units were AT and AG, accounting for 37.92% and 31.16% of the total microsatellite loci, respectively. A phylogenetic analysis showed that samples with the same altitude (Xunyangba, Ningshan country, and Zhangliangmiao, Liuba country) had a strong bootstrap value (88%), while the remaining ones shared a similar longitude. These results provided clues about the importance of longitude/altitude for the genetic diversity of *Acer miaotaiense*. This information will be useful for the conservation and improved management of this endangered species.

## 1. Introduction

*Acer miaotaiense*, an endangered species [1], is only distributed in several small and isolated regions of China’s Qingling Mountains and Bashan Mountains [2]. Since it was discovered in 1954, this species greatly diversified the genetic diversity of this region, and also provided potential scientific value for analyzing its origin and evolution. This species is an important ornamental plant, and has a wide range of medical applications in traditional Chinese medicine. Many highly bioactive compounds with good pharmacological effects are extracted from plants belonging to the genus Acer, such as flavonoids, tannins, terpenoids, and alkaloids [3]. However, this species is vulnerable to environmental changes and anthropogenic disturbance [4,5]. It was listed as a vulnerable species in a recent nationwide biodiversity report [6]. A recent genetic diversity study using RAPD markers showed that this species has a low level of genetic diversity, especially at the population level; the gene flow among populations is also low [7]. Clustering analysis showed that genetic differentiation occurred between individuals from the middle and west of the Qinling Mountains, and clustering results were inconsistent with geographical distributions [7]. Dissections of the phylogeographic and phylogenetic studies are needed for better conservation of this endangered species.

Chloroplasts (cp) are essential organelles in higher plant cells with an important role in photosynthesis and the metabolism of fatty acids, nitrogen, and internal redox signals [8,9,10]. The chloroplast genome is a typical double-stranded cyclic structure, and consists of one large single-copy region (LSC), a small single-copy region (SSC), and two inverted repeat regions (IRs) [10]. The total number of genes in plant chloroplast genomes generally ranges from 110 to 130, with a conserved genomic composition and arrangement. The main gene families include photosynthesis, self-reproduction, chloroplast transcriptional expression-related genes, and some unknown genes [2,11]. Though generally highly conserved, there are still minor changes undergone in the chloroplast genome, such as size changes, contraction and expansion of IRs, structure rearrangement, and environments [12].

Comparative analysis of the cp genome within a species might provide clues to colonization histories and population dynamics under different environments [13,14]. We herein present the whole cp genome sequence of *Acer miaotaiense* from five geographical locations using the Illumina sequencing technology. We also characterized the microsatellites detected in the genome, including the repeat types, distribution patterns, etc. Comparative sequence analysis and phylogenetic relationships were also analyzed. These results will be helpful for the conservation of this endangered species, and also to deepen our understanding of the structural diversity of the cp genome of *Acer miaotaiense* under different geographical environments.

## 2. Results and Discussion

### 2.1. Genomic Features

The complete chloroplast genome of *Acer miaotaiense* contains 19,087,438 reads and 5,726,231,340 base numbers on average (Table S1). The percentage of high base quality (>Q30, representing 99.9% base call accuracy) was quite high (Q30 > 91.67%). The GC content accounted for 36.77% of the total chloroplast genome, which is an important indicator of species affinity. All samples from five locations had a low range of GC content. This observed content was similar to other *Acer* species [11,12,13], and is slightly lower (37.88%) than that of the *Acer miaotaiense* reported in Taibai Country [2]. The total genome size was 156,238 bp on average (Table 1), which was similar to a recent report [2].

**Figure 1:**
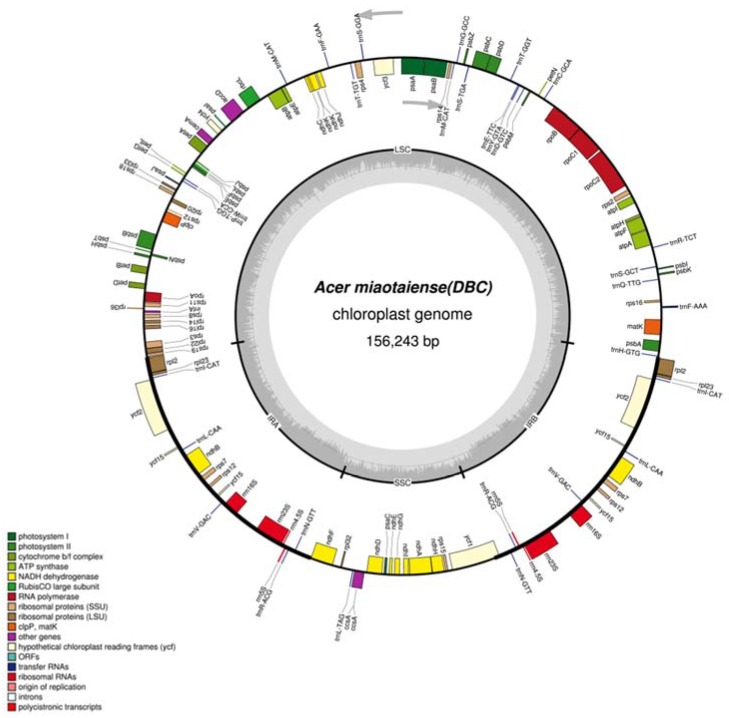
Circular gene map of *Acer miaotaiense* from the location of Dianbingchang, Meixian country (DB). Genes on the outside circle are transcribed counterclockwise, while genes on the inside circle are presented clockwise. LSC, large single copy; SSC, small single copy; INA, inverted repeat A; INB, inverted repeat B.

The genome contains four regions, including two inverted repeat regions (IRA and IRB), a small single-copy (SSC) region, and a large single-copy (LSC) region (Figure 1 and Figures S2–S4). The largest genomic component was LSC, with 86,095 bp on average, accounting for 55% of the chloroplast genome. In comparison, the IR and SSC regions accounted for 33.33% and 11.57%, respectively (on average). The largest and smallest chloroplast genomes of *Acer miaotaiense* were found in Zhangliangmiao, Liuba country (ZL) and Dianbingchang, Meixian country (DB) with lengths of 156,260 bp and 156,204 bp, respectively (Table 1). The structure of the cp genome of *Acer miaotaiense* was similar to those from the other *Acer* species, such as *Acer buergerianum* [15], *Acer morrisonense* [16], and *Acer davidii* [17], in terms of genome size and the length of the four main regions.

Across the whole chloroplast genome, there were 130 genes in total, and 92 of them were protein-coding genes (Table 2). The remaining ones were tRNA and rRNA genes, with numbers of 30 and eight, respectively. Among them, genes involved in photosynthesis and self-replication were the two dominant gene families (Table 2). There were six genes coding the subunits of ATP synthase and 11 genes associated with the subunits of NADH dehydrogenase. Five (*psaA*, *psaB*, *psaC*, *psaI*, and *psaJ*) and 15 genes (*psbA*, *psbB*, *psbC*, *psbD*, *psbE*, *psbF*, *psbH*, *psbI*, *psbJ*, *psbK*, *psbL*, *psbM*, *psbN*, *psbT*, and *psbZ*) were also identified coding the subunits of photosystem I and photosystem II, respectively. Of the 130 genes, there were five genes with unknown functions (*ycf*1, *ycf*2, *ycf*3, *ycf*4, and *ycf*15). These results showed that, for the cp genome of *Acer miaotaiense*, the number of genes and protein-coding genes is quite conserved across the five studied geographical locations. The total number of genes was slightly lower than that for some *Acer* species, such as *Acer davidii* Franch (134 genes) [17], and *Acer buergerianum* (134 genes) [15], but with a higher number of protein-coding genes. During the evolution of angiosperms, multiple gene losses remain an ongoing process [10,18,19,20]. Functional and non-functional genes from the plastid genome can be transferred to the nuclear and the mitochondrial genome [21,22]. In some parasitic species, such as those belonging to the Orobanchaceae family, gene losses can happen in the gene-encoding subunits of the genetic apparatus [23], and are not restricted to genes that are involved in photosynthesis and related pathways [24,25]. The loss of genome could serve as a low-cost strategy under disadvantageous environmental conditions by facilitating rapid genome replication [25,26,27].

**Figure 2:**
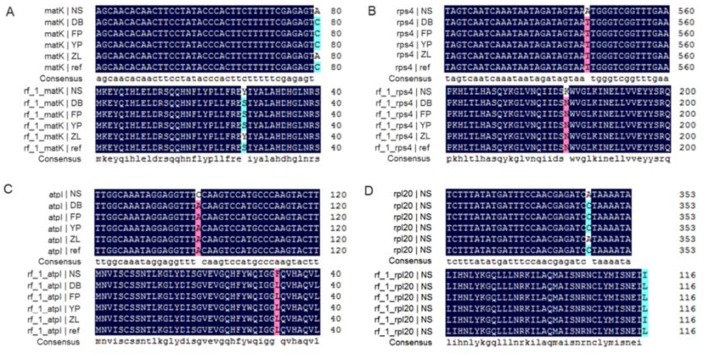
Non-synonymous mutations caused by SNPs in the coding regions of genes in the chloroplast genome of *Acer miaotaiense* from five geographical locations—Xunyangba, Ningshan country (NS), Dianbingchang, Meixian country (DB), Shiziba, Foping country (FP), Yangpigou, Taibai country (YP), and Zhangliangmiao, Liuba country (ZL). (**A**) Non-synonymous mutations caused by SNPs in the coding regions of *matK*. Upper part with highlight indicates the position of the SNP, and the lower part with highlight indicates the corresponding amino acid changes. (**B**) Non-synonymous mutations caused by SNPs in the coding regions of *rps4*. Upper part with highlight indicates the position of the SNP, and the lower part with highlight indicates the corresponding amino acid changes. (**C**) Non-synonymous mutations caused by SNPs in the coding regions of *atpI*. Upper part with highlight indicates the position of the SNP, and the lower part with highlight indicates the corresponding amino acid changes. (**D**) Non-synonymous mutations caused by SNPs in the coding regions of *rp120*. Upper part with highlight indicates the position of the SNP, and the lower part with highlight indicates the corresponding amino acid changes.

We observed SNP variations in the coding regions of five genes, including *matK*, *atpI*, *rps*4, *atpB*, and *rpl*20 (Table S2). Among these, the SNP in *atpB* was a synonymous mutation in ZL (Figure S5), and the remaining four caused non-synonymous mutations (Figure 2). The *matK* gene is involved in post-transcriptional modification [28], and regulation mechanisms relating to plant development and indirect photosynthesis [29]. It is commonly referred to as maturase-associated with several intron-containing plastid mRNAs [30]. The *atpI* gene is a membrane protein encoded by *atp* operons, and is necessary during the assembly of some ATP synthase complexes [31]. The *rps*4 gene belongs to the family of a small subunit of ribosome, and involves the decoding of genetic information during translation [32]. In contrast, *rpl*20 belongs to a family of a large subunit of ribosome, and catalyzes peptide bond formation [33]. Notably, all SNP variations causing non-synonymous mutation of the aforementioned genes occurred in at least one location, and they all occurred in Xunyangba, Ningshan country (NS). The surrounding environment is more disadvantageous, compared with other locations, in terms of artificial disturbances, diseases, and pests. These results provided clues regarding local adaptions under environments via altering post-transcriptional modification, photosynthesis, and self-replication. Chloroplast plays vital roles in the biosynthesis of many essential metabolites, such as amino acids, fatty acids, vitamins, etc. These metabolites are important for the response to diverse biotic (e.g., pathogens) and abiotic stresses (heat, drought, salt, etc.) [9,12,34].

### 2.2. Comparative Genomic Analysis

**Figure 3:**
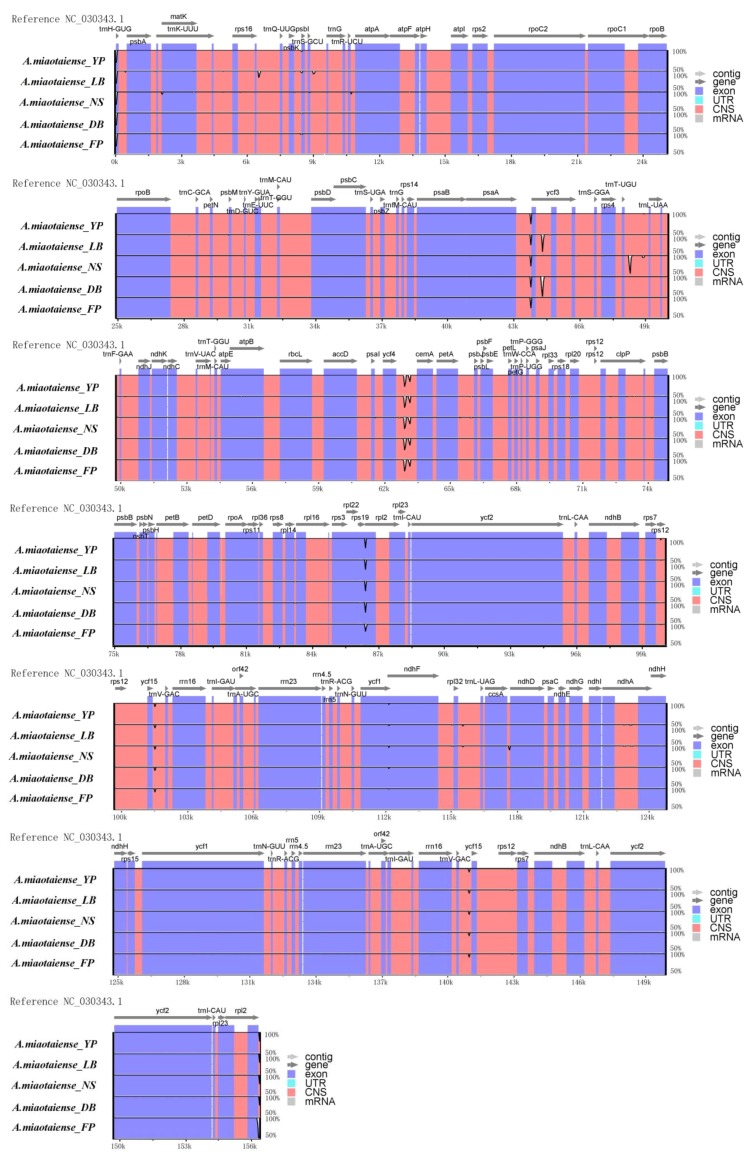
Sequence alignment of the chloroplast genome features of *Acer miaotaiense* from five geographical locations. The alignment was done using the mVISTA program, and the reference chloroplast genome was NC_030343.1. The vertical scale represents the degree of identity, which ranged from 50% to 100%. Coding and non-coding regions are marked in blue and red, respectively. Black arrows indicate the position and direction of each gene.

Comparative analysis of cp genomes is crucial in understanding the diversity and evolution of a plant under different environments [12,35]. The sequence identity was quite high across the five geographical locations (Figure 3). In addition, both the gene order and number were highly conserved in the cp genomes across the five geographical locations. The difference across the length of the chloroplast genome was mainly caused by variance in the length of LSCs. The majority of the variances occurred in the conserved non-coding regions. These results indicated the non-coding regions were less conserved, compared to coding regions, which was also found in some other species, such as *Cerasus humilis* [36], *Talinum paniculatum* [37], and *Heimia myrtifolia* [38].

### 2.3. IR Expansion and Contraction

**Figure 4:**
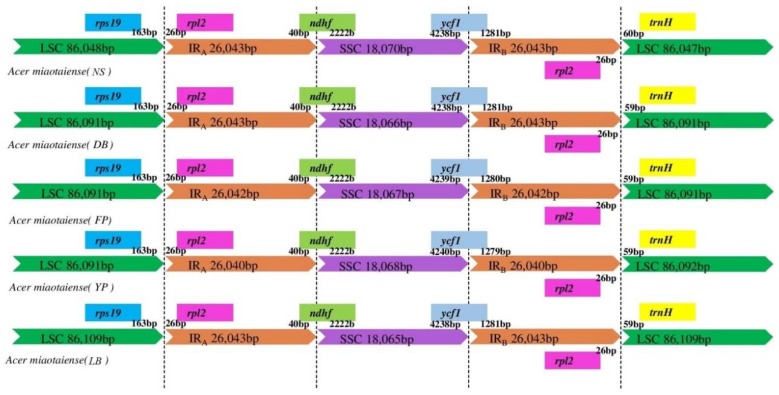
Comparison of the LSC, IR, SSC, and LSC boundaries of the chloroplast genome of *Acer miaotaiense* from five geographical locations. The adjacent border genes are indicated by boxes with gene names above or below the main line. Gaps between the ends of boundaries and adjacent genes were indicated in bps above the main line.

After comparison of the LSC, IR, SSC, and LSC boundaries of the cp genome of *Acer miaotaiense*, we found that the IR contraction and expansion were highly similar across the five geographical locations (Figure 4). The *ycf*1 gene was located across the SSC and IRB regions, and the starting position was different in Shiziba, Foping country (FP) and Yangpigou, Taibai country (YP) compared with the other three locations. The *trnH* gene was located in the LSC region, and was shifted 1 bp to the right, compared with the other locations. These results showed that, with the species of *Acer miaotaiense*, the genomic structure was highly conserved, though genomic length variation can be found in the LSC and SSC boundary regions, as reported in other species [36,38,39].

### 2.4. Microsatellite Detection Analysis

**Figure 5:**
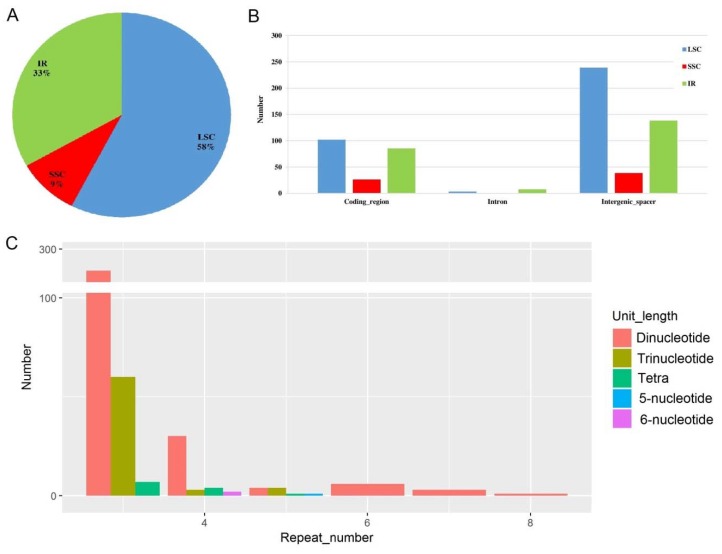
Characterization of microsatellite loci detected in the chloroplast genome of *Acer miaotaiense* from five geographical locations. (**A**) Percentage of microsatellite loci in the IR, LSC, and SSC regions. (**B**) Number and distribution of microsatellite loci in the protein-coding regions, introns, and intergenic spacers in the LSC, SSC, and IR regions. (**C**) Number and distribution of microsatellite loci with different repeat numbers.

A total of 415 microsatellite loci (or simple sequence repeats, SSRs) were identified in the cp genome of *Acer miaotaiense* (Figure 5). Among them, the largest number of loci was identified in the LSC region across five geographical locations, accounting for 58% of the total microsatellite loci, followed by the IR regions (Figure 5A). There were 102, 26, and 85 microsatellite loci identified in the protein-coding regions of genes in the LSC, SSC, and IR regions, respectively. No microsatellite loci were found in the intron regions of genes in the cp genome (Figure 5B). The dominant microsatellite types were dinucleotide and trinucleotide with repeat number of three (Figure 5C).

**Figure 6:**
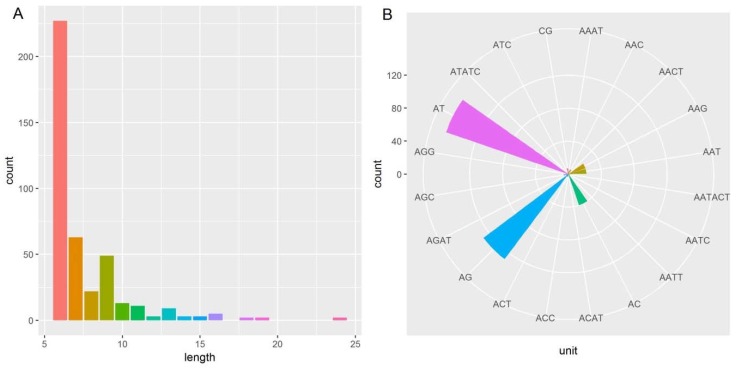
Number of microsatellite loci with different repeat lengths (**A**) and repeat units (**B**) detected in the chloroplast genome of *Acer miaotaiense*.

The length of repeats ranged from six to 24, with an average value of 7.5. The dominant repeat length was six (54.83%), followed by seven (15.22%), and nine (11.84%), as shown in Figure 6A. Up to 20 repeat types were detected across the cp genome of *Acer miaotaiense*. Among these, AT and AG were the two dominant repeat types, accounting for 37.92% and 31.16% of the total microsatellite loci, respectively (Figure 6B).

In over 400 microsatellite loci identified, the number of loci in the LSC, SSC, and IR regions across different geographical locations (DB, YP, ZL, and NS) was highly conserved, with 239, 38, and 138, respectively. For the location of FP, 240, 38, and 137 microsatellite loci were detected in the LSC, SSC, and IR regions, respectively. Similar patterns were also reported in other species, such as *Cerasus humilis* [36], *Arabis stellari* [40], and *Paeonia ostii* [41]. However, though the total number of microsatellites was highly conserved, polymorphisms might still be observed in certain loci, which could be helpful in population and evolutionary studies [42,43,44].

### 2.5. Phylogenetic Analysis

**Figure 7:**
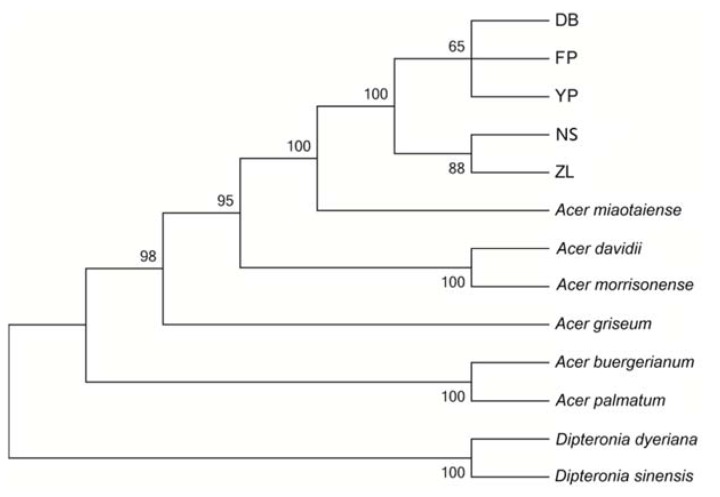
Phylogenetic relationships based on 61 common coding genes in the chloroplast genomes of *Acer miaotaiense* species with two outgroup species (*Dipteronia dyeriana* and *Dipteronia sinensis*) using the maximum-likelihood approach.

To analyze the phylogenetic relationships of *Acer miaotaiense*, a total of 61 common coding genes of the cp genomes from eight species were analyzed, including two outgroup species (*Acer miaotaiense*, *Acer davidii*, *Acer morrisonense*, *Acer griseum*, *Acer buergerianum*, *Acer palmatum*, *Dipteronia dyeriana*, and *Dipteronia sinensis*), shown in Figure 7. The phylogenetic tree demonstrated that the *Acer miaotaiense* from the five geographical locations were closely clustered together with a strong bootstrap value of 100%. All *Acer* species formed the main groups with strong bootstrap values, excluding the two outgroup species (*Dipteronia dyeriana* and *Dipteronia sinensis*). Among the five locations, DB, FP, and YP were located at approximately the same longitude, while the locations of NS and ZL had the same altitude (1224 m) with a strong bootstrap value (88%). These results indicated that differences in geographical environment, especially longitude, might be responsible for the genetic diversity of *Acer miaotaiense*. Also, sequencing depth, annotation methods [45], gene losses, pseudogenizations, and exceptional gene gains could also possibly affect the result [10,19].

## 3. Materials and Methods

### 3.1. Plant Materials DNA Isolation

Fresh and young leaves of *Acer miaotaiense* were collected from five geographic locations in Shaanxi, China. The DB, YP, and FP regions had a similar longitude. DB was located in the northernmost area of the five regions (Table 3). YP had the highest altitude, and FP had the lowest altitude. In addition, the climate in YP was more complicated, and the distributions of *Acer miaotaiense* suffered from severe artificial deforestation activities, resulting in serious damage. DB was located in the middle of the Qingling Mountains, where minor insects could be observed. FP had a moderate climate with an average temperature of 25 °C in June. The largest distance (156 km) was found between ZL and NS, while the closest distance was between DB and YP (around 3 km). Total genomic DNA was isolated according to the manufacturer’s protocol using the HP PlantDNA Kit D2485-01 (Omega Bio-Tek, Santa Clara, CA, USA).

### 3.2. Chloroplast Genome Sequencing, Assembly, and Annotation

After quantification and qualification, a paired-end library was constructed, and high-throughput sequencing was performed using the Illumina Hiseq 2500 platform (Lemont, IL, USA). After cleaning the raw data, a total of 28.5 Gb of high-quality clean data (≥Q30 values were higher than 89.76% for all samples) was retained. The complete chloroplast genome of *Acer miaotaiense* was assembled using the NOVOPlasty software [46], according to the standard default parameters. The circular map of the fully annotated genome was drawn in OGDRAW v1.2 [47]. All five cp genomes were also deposited in the GenBank database.

### 3.3. Comparative Genome Analysis

Comparative analysis of the chloroplast genome of the members of the genus Acer was done using the mVISTA program [48], based on the LAGAN alignment strategy. One genome in the same family as *Acer miaotaiense* was used as the reference (GenBank Accession No. NC_030343.1). Differences in the types and gene sizes of the IR, LSC, and SSC border regions among related species were also analyzed.

### 3.4. Microsatellite Detection Analysis

Microsatellites, also known simple sequence repeats (SSRs), were analyzed using PHOBOS 3.3.12 (http://www.rub.de/ecoevo/cm/cm_phobos.htm). A more detailed characterization of the detected microsatellite loci in different regions of IR, LSC, and SSC was carried out, as well as the repeat types being analyzed.

### 3.5. Phylogenetic Analysis

The chloroplast genome sequences of eight species (*Acer miaotaiense*, *Acer davidii*, *Acer morrisonense*, *Acer griseum*, *Acer buergerianum*, *Acer palmatum*, *Dipteronia dyeriana*, and *Dipteronia sinensis*) were downloaded from NCBI (https://www.ncbi.nlm.nih.gov/). After searching all common coding genes, a total of 61 common genes (*accD*, *atpA*, *atpB*, *atpE*, *atpF*, *atpH*, *atpI*, *ccsA*, *cemA*, *ndhA*, *ndhB*, *ndhC*, *ndhD*, *ndhE*, *ndhG*, *ndhH*, *ndhJ*, *ndhK*, *petA*, *petB*, *petD*, *petG*, *petL*, *petN*, *psaA*, *psaB*, *psaI*, *psbA*, *psbB*, *psbC*, *psbD*, *psbE*, *psbF*, *psbH*, *psbJ*, *psbM*, *psbN*, *psbT*, *rbcL*, *rpl*14, *rpl*16, *rpl*2, *rpl*20, *rpl*22, *rpl*23, *rpl*32, *rpl*33, *rpoA*, *rpoC*1, *rpoC*2, *rps*11, *rps*14, *rps*15, *rps*18, *rps*19, *rps*3, *rps*7, *rps*8, *ycf*2, *ycf*3, and *ycf*4) were retained for the construction of phylogenetic trees. The reference genome was *Acer miaotaiense* (GenBank accession no. NC_030343.1). Sequence alignment was done in MAFFT v7 [49]. The maximum-likelihood (ML) tree was reconstructed using MEGA v6 [50], and the number of bootstrap replications was 500 for the phylogeny test.

## 4. Conclusions

In this study, we reported and compared the chloroplast genome of *Acer miaotaiense* from five ecological regions in the Qingling and Mashan Regions of China. We observed four genes with non-synonymous mutations involving post-transcriptional modification, photosynthesis, and self-replication. A total of 415 microsatellite loci were identified, which are helpful in developing polymorphic molecular markers. Our phylogenetic analysis showed that samples with the same altitude or similar longitude were more closely related, providing clues to the importance of longitude/altitude for the genetic diversity of *Acer miaotaiense*. This information will be useful for the conservation and improved management of this endangered species.

## Figures and Tables

**Table 1:** Summary of the chloroplast genome features of *Acer miaotaiense*.

Type	DB	YG	ZL	NS	FP	Average
Size (bp)	156,243	156,239	156,260	156,204	156,242	156,238
LSC length (bp)	86,091	86,093	86,109	86,048	86,136	86,095
SSC length (bp)	18,068	18,068	18,067	18,072	18,068	18,069
IR length (bp)	52,084	52,078	52,084	52,084	52,038	52,074
Number of gene	130	130	130	130	130	130
Protein-coding genes	92	92	92	92	92	92
tRNA genes	30	30	30	30	30	30
rRNA genes	8	8	8	8	8	8

Note: DB, Dianbingchang, Meixian country; YP, Yangpigou, Taibai country; ZL, Zhangliangmiao, Liuba country; NS, Xunyangba, Ningshan country; FP, Shiziba, Foping country.

**Table 2:** List of the genes in the chloroplast genome features of *Acer miaotaiense*.

Gene Functions	Gene Family	Gene Names
Photosynthesis	Subunits of ATP synthase	*atpA*, *atpB*, *atpE*, *atpF*, *atpH*, *atpI*
	Subunits of NADH dehydrogenase	*ndhA*, *ndhB*, *ndhC*, *ndhD*, *ndhE*, *ndhF*, *ndhG*, *ndhH*, *ndhI*, *ndhJ*, *ndhK*
	Subunits of cytochrome	*petA*, *petB*, *petD*, *petG*, *petL*, *petN*
	Subunits of photosystem I	*psaA*, *psaB*, *psaC*, *psaI*, *psaJ*
	Subunits of photosystem II	*psbA*, *psbB*, *psbC*, *psbD*, *psbE*, *psbF*, *psbH*, *psbI*, *psbJ*, *psbK*, *psbL*, *psbM*, *psbN*, *psbT*, *psbZ*
	Subunit of rubisco	*rbcL*
Other genes	Subunit of Acetyl-CoA-carboxylase	*accD*
	c-type cytochrome synthesis gene	*ccsA*
	Envelop membrane protein	*cemA*
	Protease	*clpP*
	Translational initiation	*infA*
	Maturase	*matK*
Self-replication	Large subunit of ribosome	*rpl*2, *rpl*14, *rpl*16, *rpl*20, *rpl*22, *rpl*23, *rp*32, *rpl*33, *rpl*36
	DNA dependent RNA polymerase	*rpoA*, *rpoB*, *rpoC*1, *rpoC*2
	Small subunit of ribosome	*rps*2, *rps*3, *rps*4, *rps*7, *rps*8, *rps*11, *rps*12, *rps*14, *rps*15, *rps*16, *rps*18, *rps*19
	rRNA Genes	*rrn*4.5*S*, *rrn*5*S*, *rrn*16*S*, *rrn*23*S*
	tRNA Genes	*trnC-GCA*, *trnD-GTC*, *trnE-TTC*, *trnF-AAA*, *trnF-GAA*, *trnG-GCC*, *trnH-GTG*, *trnI-CAT*, *trnL-CAA*, *trnL-TAG*, *trnM-CAT*, *trnN-GTT*, *trnP-TGG*, *trnQ-TTG*, *trnR-ACG*, *trnR-TCT*, *trnS-GCT*, *trnS-GGA*, *trnS-TGA*, *trnT-GGT*, *trnT-TGT*, *trnV-GAC*, *trnW-CCA*, *trnY-GTA*
Unknown function	Conserved open reading frames	*ycf*1, *ycf*2, *ycf*3, *ycf*4, *ycf*15

**Table 3:** Detailed information of the five geographic locations of *Acer miaotaiense*.

Code	Location	Longitude	Latitude	Altitude (m)
DB	Dianbingchang, Meixian country	E107°41′54″	N34°4′19″	1427
YP	Yangpigou, Taibai country	E107°40′54″	N34°1′56″	1506
ZL	Zhangliangmiao, Liuba country	E106°50′10″	N33°40′58″	1224
NS	Xunyangba, Ningshan country	E108°30′49″	N33°33′40″	1224
FP	Shiziba, Foping country	E107°52′31″	N33°29′14″	945

## Supplementary Materials

The following are available online at http://www.mdpi.com/1420-3049/23/7/1740/s1, Figure S1: Circular gene map of *Acer miaotaiense* from the location of YP. Genes at the outside circle are transcribed counterclockwise, while genes inside of the circle are presented clockwise. LSC, large single copy; SSC, small single copy; INA, inverted repeat A; INB, inverted repeat B, Figure S2: Circular gene map of *Acer miaotaiense* from the location of ZL. Genes at the outside circle are transcribed counterclockwise, while genes inside of the circle are presented clockwise. LSC, large single copy; SSC, small single copy; INA, inverted repeat A; INB, inverted repeat B, Figure S3: Circular gene map of *Acer miaotaiense* from the location of NS. Genes at the outside circle are transcribed counterclockwise, while genes inside of the circle are presented clockwise. LSC, large single copy; SSC, small single copy; INA, inverted repeat A; INB, inverted repeat B, Figure S4: Circular gene map of *Acer miaotaiense* from the location of FP. Genes at the outside circle are transcribed counterclockwise, while genes inside of the circle are presented clockwise. LSC, large single copy; SSC, small single copy; INA, inverted repeat A; INB, inverted repeat B, Figure S5: Variation in the gene of *atpB* across five locations. (A) SNP variation position in the gene of *atpB*. (B) Amino acids where the SNP occurred, Table S1: Sequencing quality of *Acer miaotaiense* from five regions. Table S2. Detailed information on the detected SNPs in the chloroplast genome at five geographical locations.
