# Supplementary material for: Characterization of the Chloroplast Genome Sequence of Acer miaotaiense: Comparative and Phylogenetic Analyses
Source: Molecules. 2018 Jul 17;23(7):1740. doi: 10.3390/molecules23071740 (PMC6099587; doi:10.3390/molecules23071740)
Supplement: Supplementary file 1 [file molecules-23-01740-s001.pdf]

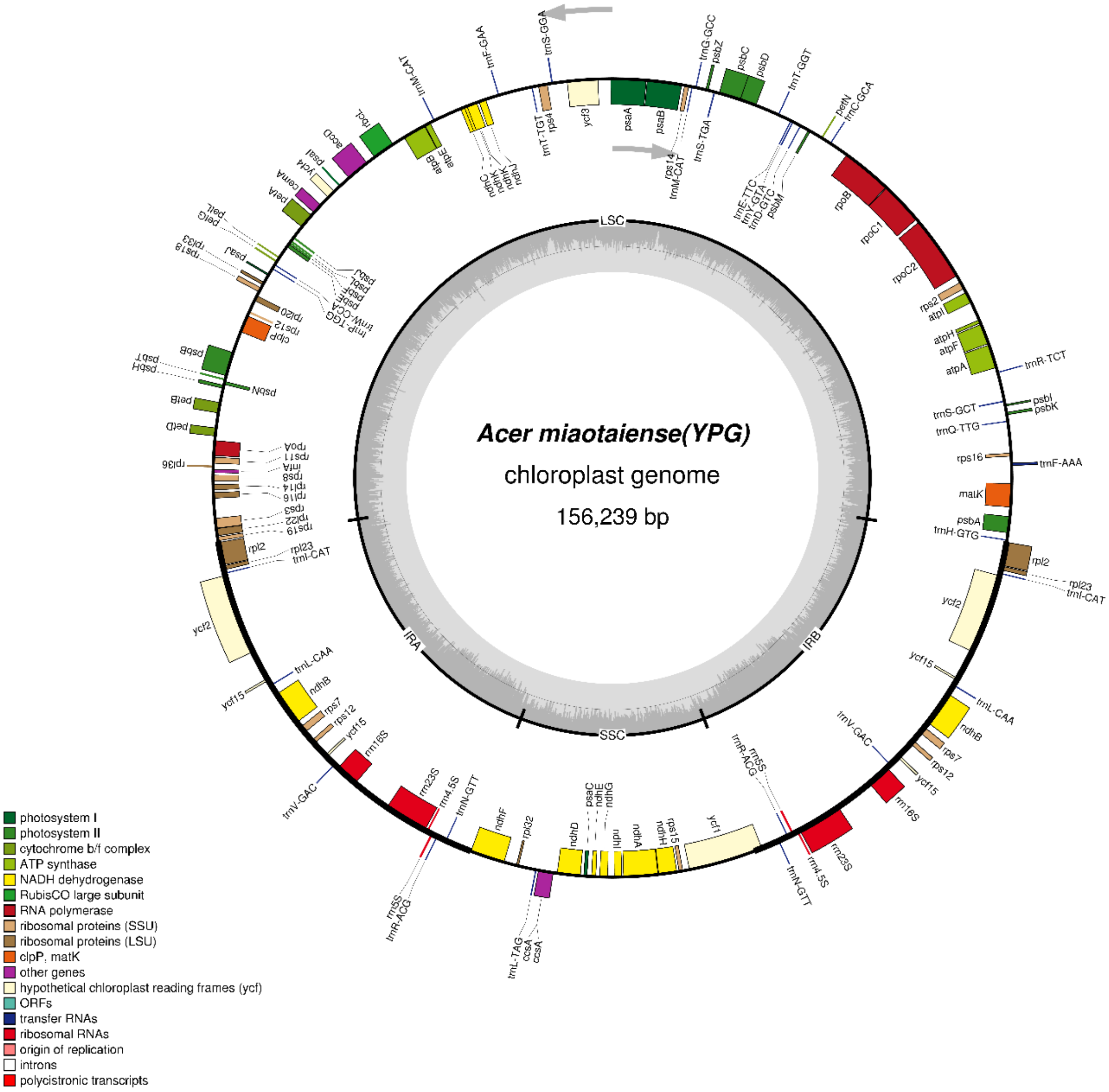
Acer miaotaiense(YPG)
chloroplast genome
156,239 bp
LSC
SSC
IRA
IRB
photosystem I
photosystem II
cytochrome b/f complex
ATP synthase
NADH dehydrogenase
RubisCO large subunit
RNA polymerase
ribosomal proteins (SSU)
ribosomal proteins (LSU)
clpP, matK
other genes
hypothetical chloroplast reading frames (ycf)
ORFs
transfer RNAs
ribosomal RNAs
origin of replication
introns
polycistronic transcripts

Figure S1

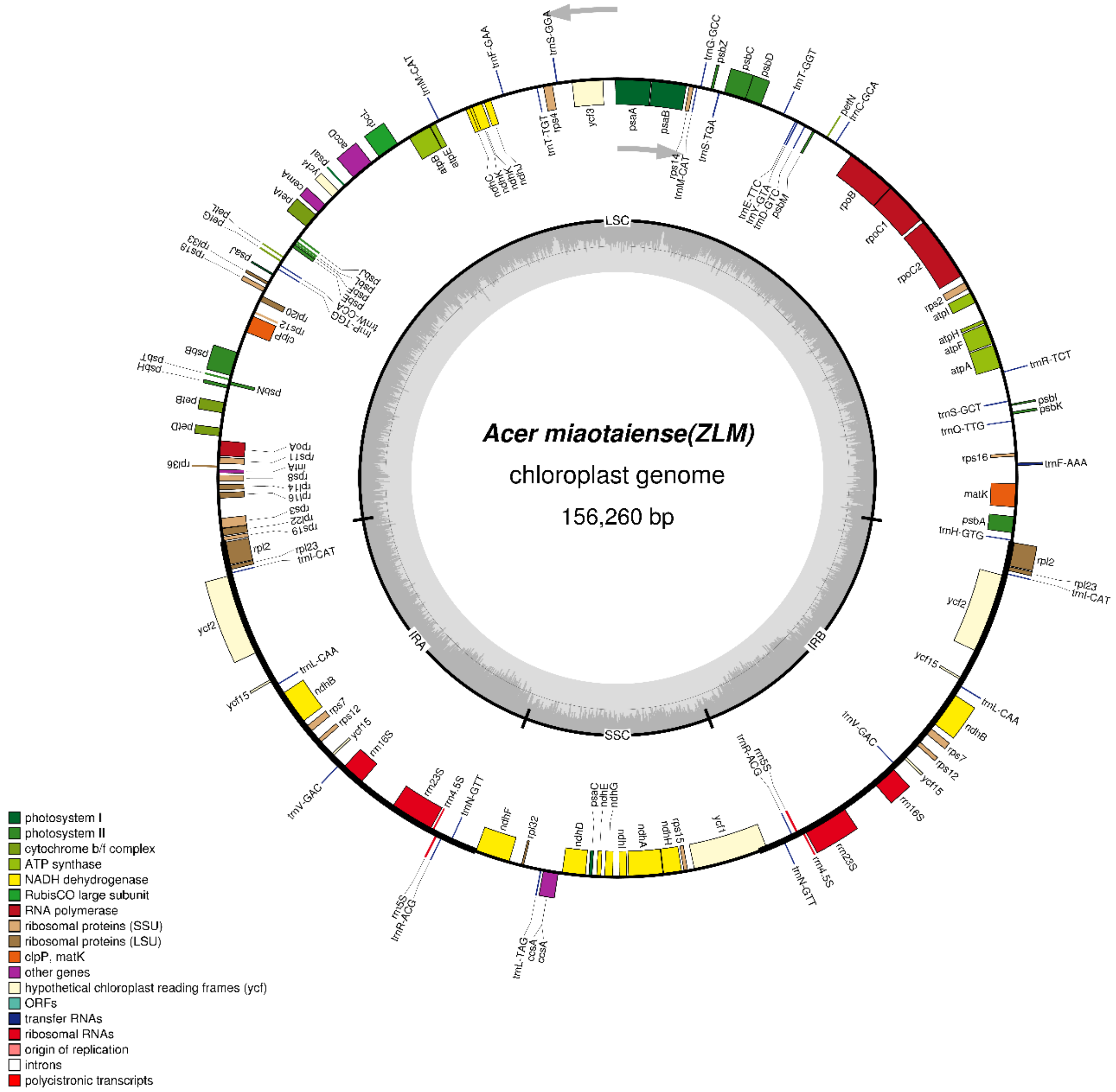
Acer miaotaiense(ZLM)
chloroplast genome
156,260 bp
LSC
IRA
IRB
SSC
photosystem I
photosystem II
cytochrome b/f complex
ATP synthase
NADH dehydrogenase
RubisCO large subunit
RNA polymerase
ribosomal proteins (SSU)
ribosomal proteins (LSU)
clpP, matK
other genes
hypothetical chloroplast reading frames (ycf)
ORFs
transfer RNAs
ribosomal RNAs
origin of replication
introns
polycistronic transcripts

Figure S2

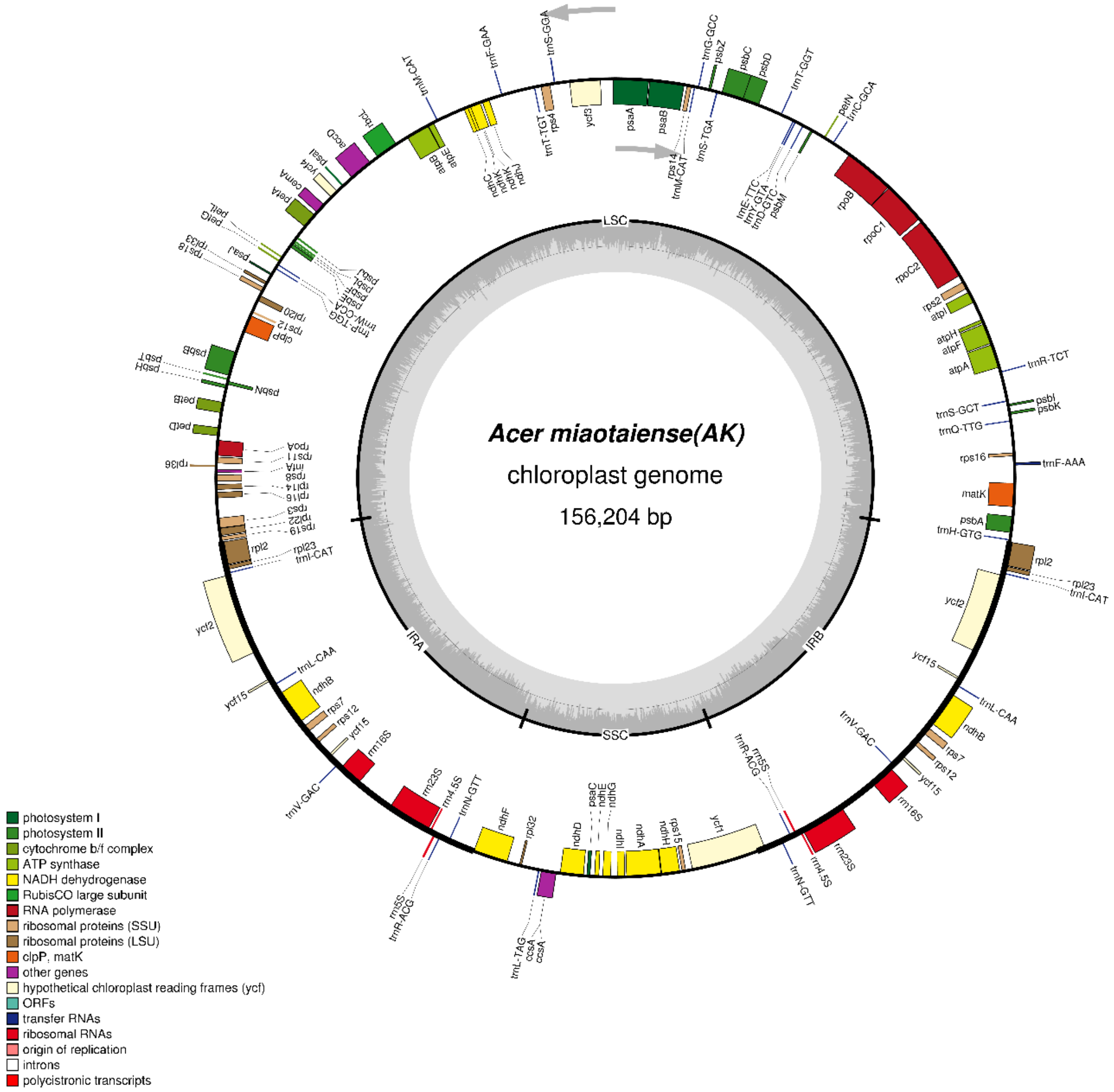

Acer miaotaiense(AK)
chloroplast genome
156,204 bp
LSC
SSC
IRA
IRB
photosystem I
photosystem II
cytochrome b/f complex
ATP synthase
NADH dehydrogenase
RubisCO large subunit
RNA polymerase
ribosomal proteins (SSU)
ribosomal proteins (LSU)
clpP, matK
other genes
hypothetical chloroplast reading frames (ycf)
ORFs
transfer RNAs
ribosomal RNAs
origin of replication
introns
polycistronic transcripts

Figure S3

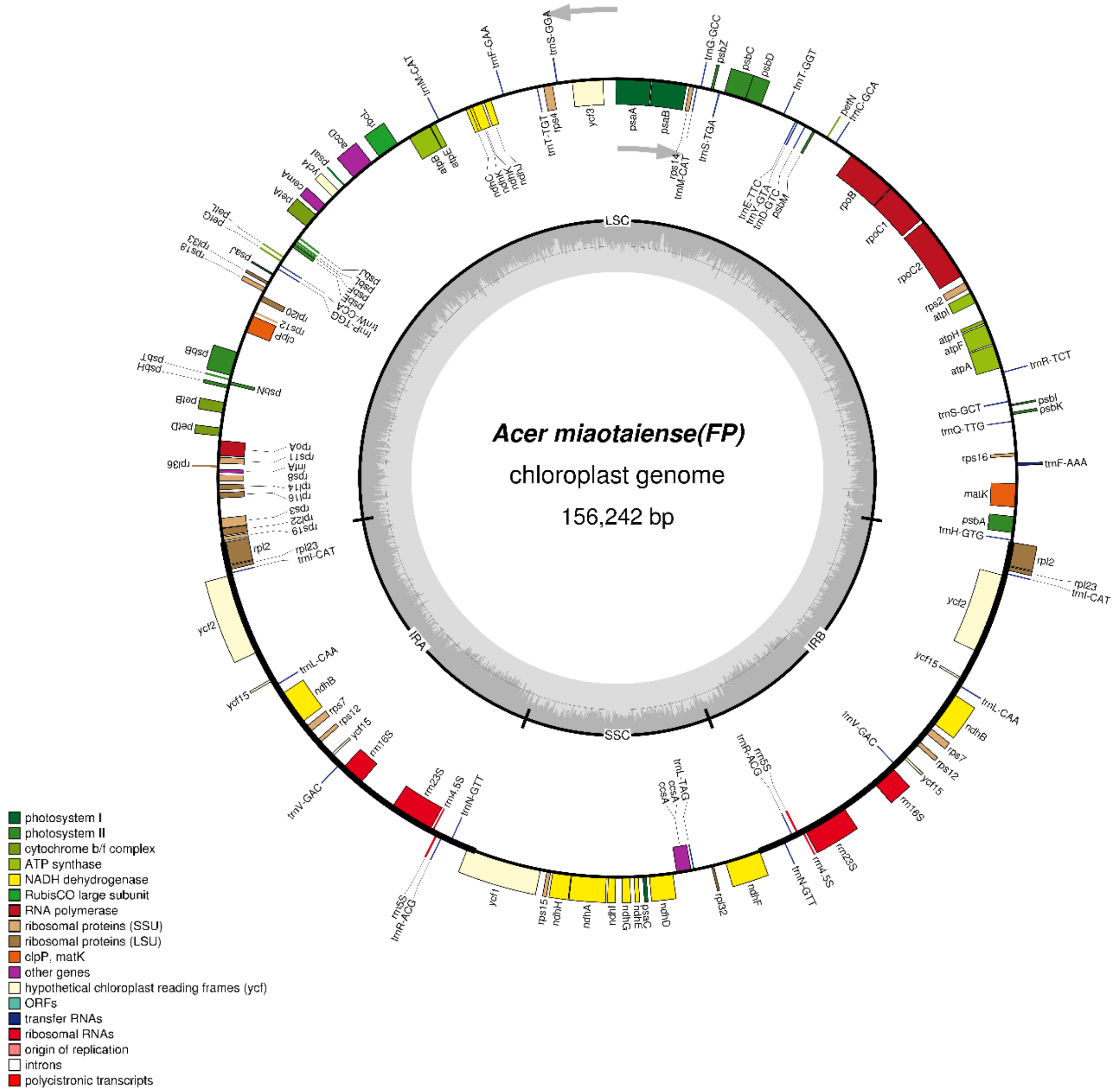
Acer miaotaiense(FP)
chloroplast genome
156,242 bp
LSC
IRB
SSC
IRA
photosystem I
photosystem II
cytochrome b/f complex
ATP synthase
NADH dehydrogenase
RubisCO large subunit
RNA polymerase
ribosomal proteins (SSU)
ribosomal proteins (LSU)
clpP, matK
other genes
hypothetical chloroplast reading frames (ycf)
ORFs
transfer RNAs
ribosomal RNAs
origin of replication
introns
polycistronic transcripts

Figure S4

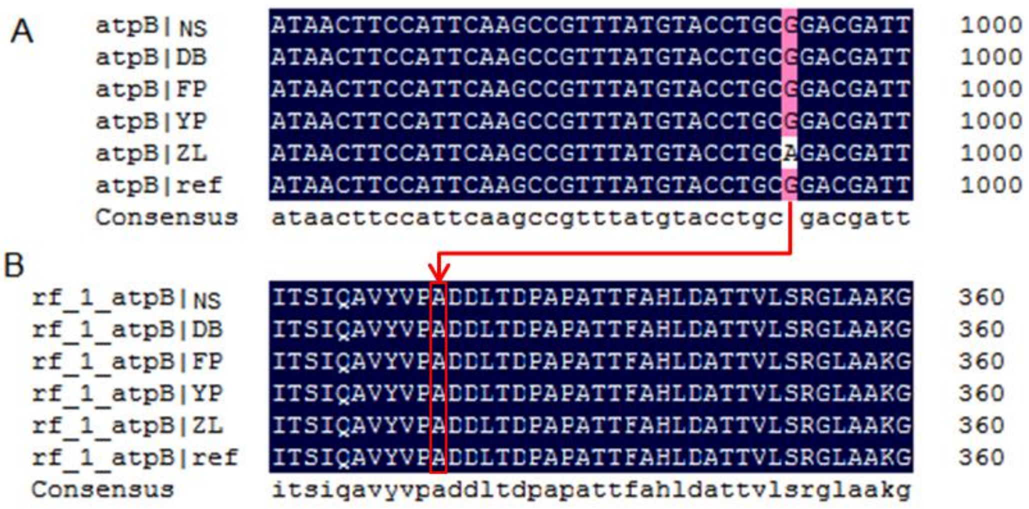

Figure S5

Table S1. Sequencing quality of *Acer miaotaiense* from five regions.

Samples	BRD-ID	Read Number	Base Number	GC Content	≥Q30
YPG	R01	19,350,980	5,805,294,000	36.78%	89.76%
ZLM	R02	18,853,398	5,656,019,400	36.36%	90.74%
AK	R03	18,094,930	5,428,479,000	36.88%	93.04%
DBC	R04	20,218,759	6,065,627,700	37.20%	93.24%
FP	R05	18,919,122	5,675,736,600	36.63%	91.59%
Average	-	19,087,438	5,726,231,340	36.77%	91.67%

Table S2. Detailed information of the detected SNPs in the chloroplast genome at five geographical locations. .

SNP	Location	position	gene	SNP	Location	position	gene	SNP	Location	position	gene	SNP	Location	position	gene	SNP	Location	position	gene	SNP	species	position	gene
t	NS	73744	matK	g	DB	73734	matK	g	FP	73711	matK	g	YP	73730	matK	t	ZL	73734	matK	g	ref	3532	matK
g	NS	86091	atpI	t	DB	86077	atpI	t	FP	86054	atpI	t	YP	86073	atpI	t	ZL	86093	atpI	t	ref	15873	atpI
t	NS	117326	rps4	a	DB	117312	rps4	a	FP	117289	rps4	a	YP	117308	rps4	a	ZL	117328	rps4	a	ref	47082	rps4
c	NS	125832	atpB	c	DB	125870	atpB	c	FP	125847	atpB	c	YP	125866	atpB	t	ZL	125885	atpB	c	ref	55640	atpB
t	NS	140451	rpl20	g	DB	140488	rpl20	g	FP	140465	rpl20	g	YP	140484	rpl20	t	ZL	140504	rpl20	g	ref	70621	rpl20
